# Numb is not a critical regulator of Notch-mediated cell fate decisions in the developing chick inner ear

**DOI:** 10.3389/fncel.2015.00074

**Published:** 2015-03-12

**Authors:** Mark Eddison, Sara J. Weber, Linda Ariza-McNaughton, Julian Lewis, Nicolas Daudet

**Affiliations:** ^1^Howard Hughes Medical Institute, Janelia Research CampusAshburn, VA, USA; ^2^Ear Institute, University College LondonLondon, UK; ^3^Haematopoietic Stem cell Laboratory, Cancer Research UK, London Research InstituteLondon, UK; ^4^Formerly of Vertebrate Development Laboratory, Cancer Research UKLondon, UK

**Keywords:** Numb, asymmetric cell division, Notch, lateral inhibition, inner ear, chick, hair cells

## Abstract

The Notch signaling pathway controls differentiation of hair cells and supporting cells in the vertebrate inner ear. Here, we have investigated whether Numb, a known regulator of Notch activity in *Drosophila*, is involved in this process in the embryonic chick. The chicken homolog of Numb is expressed throughout the otocyst at early stages of development and is concentrated at the basal pole of the cells. It is asymmetrically allocated at some cell divisions, as in *Drosophila*, suggesting that it could act as a determinant inherited by one of the two daughter cells and favoring adoption of a hair-cell fate. To test the implication of Numb in hair cell fate decisions and the regulation of Notch signaling, we used different methods to overexpress Numb at different stages of inner ear development. We found that sustained or late Numb overexpression does not promote hair cell differentiation, and Numb does not prevent the reception of Notch signaling. Surprisingly, none of the Numb-overexpressing cells differentiated into hair cells, suggesting that high levels of Numb protein could interfere with intracellular processes essential for hair cell survival. However, when Numb was overexpressed early and more transiently during ear development, no effect on hair cell formation was seen. These results suggest that in the inner ear at least, Numb does not significantly repress Notch activity and that its asymmetric distribution in dividing precursor cells does not govern the choice between hair cell and supporting cell fates.

## Introduction

In many tissues, the Notch pathway mediates lateral inhibition and thereby governs cell diversification: cells expressing high levels of Notch ligands activate Notch in neighboring cells and in this way force them to adopt a different fate. This process has been well studied in the developing mechanosensory bristles of *Drosophila*, and parallels between these insect sensillae and the mechanosensory epithelia of the vertebrate inner ear have guided our understanding of vertebrate ear development, and in particular of the role of Notch signaling in this process (Adam et al., 1998; Eddison et al., 2000).

Each insect bristle derives from a single sense-organ precursor (SOP) cell that undergoes a stereotyped sequence of asymmetric cell divisions. After each division, the two daughter cells adopt distinct cell fates that depend on the levels of Notch activation that they experience (Hartenstein and Posakony, 1990). In principle, the difference between the two cells could be generated through a feedback loop based on the ability of activated Notch to inhibit expression of Notch ligands (Chitnis, 1995; Heitzler et al., 1996). Such an effect, if present, means that neighboring cells will interact competitively, each tending to inhibit the other; and mathematical modeling shows that this competitive interaction can be sufficient to amplify any initial small difference between the cells and so to drive them along distinct paths of differentiation (Collier et al., 1996). In many cases, however, and in particular in insect bristle development, it appears that the interaction between sister cells is strongly biased from the outset by modulators of Notch signaling, such as Numb or Neuralized, which are asymmetrically localized in the parent cell and unequally distributed to the daughter cells after mitosis (see for reviews Roegiers and Jan, 2004; Schweisguth, 2004). The daughter cell that receives the favorable inheritance wins the competition and delivers overwhelming inhibition to its sister (and to other neighbors). In this paper, we focus on one of these modulators, Numb, and examine its role in the development of the sensory patches in the vertebrate inner ear. Does it act there, as it does in the insect bristle, to control choices of cell fate?

The *numb* gene takes its name from the *Drosophila* phenotype. Flies with loss-of-function mutations in this gene lack functional sensillae, because the daughters and grand-daughters of the SOP cells fail to diversify correctly: in place of a bristle consisting of a shaft cell, a socket cell, a neuron, and a neural sheath cell, the SOP generates a cluster of four socket cells. Conversely, artificial overexpression of *numb* can bias cell fate choices in an opposite way, causing the SOP cell (in extreme cases) to generate a cluster of four neurons (Uemura et al., 1989; Rhyu et al., 1994; Knoblich et al., 1995). The Numb gene product is a membrane-associated protein that has a conserved phosphotyrosine binding (PTB) domain and several conserved protein-protein interaction motifs in its proline-rich C-terminal region, representing binding sites for alpha-adaptin and a variety of other components of the machinery of clathrin-mediated endocytosis and ubiquitylation (Santolini et al., 2000). In the SOP lineage, Numb is asymmetrically localized at mitosis and influences cell fate decisions by reducing Notch activity in the cell that inherits it after an asymmetrical cell division (Frise et al., 1996; Guo et al., 1996; Spana and Doe, 1996; Bhalerao et al., 2005). Numb may exert its inhibitory effect by direct binding to the cytoplasmic domain of Notch (Guo et al., 1996; Zhong et al., 1996), by promoting the internalization and/or degradation of cell-surface Notch protein (Santolini et al., 2000; Berdnik et al., 2002; McGill and McGlade, 2003), by interfering with positive modulators of Notch signaling such as the transmembrane protein Sanpodo (O'Connor-Giles and Skeath, 2003; Hutterer and Knoblich, 2005; Couturier et al., 2012; Cotton et al., 2013), or by a combination of these actions.

The Notch pathway is a critical regulator of inner ear development, acting at different stages and through different ligands to control the differentiation of multiple cell types (Kiernan, 2013). Lateral inhibition regulates the production of otic neuroblasts at early stages of ear development (Adam et al., 1998; Haddon et al., 1998; Abello et al., 2007; Daudet et al., 2007), and controls hair cells vs. supporting cell fate decisions within the embryonic sensory patches (Adam et al., 1998; Lanford et al., 1999; Riley et al., 1999; Zine et al., 2000; Daudet and Lewis, 2005; Chrysostomou et al., 2012). The nascent hair cells express several Notch ligands: Delta1-like 1 (Dll1), Delta-like 3 (Dll3), and Serrate2/Jagged2 (Jag2) and activate Notch in their neighbors, which become supporting cells. The puzzling feature of the system is that the progenitor and supporting cells themselves express a Notch ligand, Jagged1 (Jag1, also known as Serrate1 in chick), which is positively regulated by Notch, a process defined as “lateral induction” (Adam et al., 1998; Lewis, 1998; Eddison et al., 2000). Jag1 contributes to the maintenance of Notch activity within progenitor cells (Neves et al., 2011), and this early phase of Notch activity is required for the maintenance, but not the initial specification, of the prosensory regions (Kiernan et al., 2001, 2006; Tsai et al., 2001; Brooker, 2006; Daudet et al., 2007; Hartman et al., 2010; Basch et al., 2011; Yamamoto et al., 2011). Although the levels of Notch activity elicited by Jag1 are thought to be relatively low compared to those resulting from Dll1 signaling (Petrovic et al., 2014), they still provide a potential obstacle to hair cell differentiation. Furthermore, direct contacts between immature hair cells or between immature hair cells and Dll1-expressing cells occur at least transiently during the development of the sensory epithelia (Goodyear and Richardson, 1997; Chrysostomou et al., 2012). How then, during normal development, do the nascent hair cells overcome Notch activation? In a previous study (Eddison et al., 2000), we reported that chicken Numb is expressed in the embryonic inner ear, and that its distribution makes it a plausible candidate to facilitate hair cell fate decisions. Because hair cells and supporting cells are derived from a common progenitor (Fekete et al., 1998; Lang and Fekete, 2001), they may perhaps be generated through asymmetric cell divisions analogous to those occurring in the insect bristle lineage.

Here, we have analyzed Numb expression pattern during chick inner ear development and have found that Numb is indeed sometimes inherited asymmetrically by the daughters of dividing precursor cells in the sensory patches. To test whether this is functionally significant, we have used *in ovo* electroporation of plasmid DNA to raise the levels of expression of Numb and analyzed the effects on hair cell fate decisions and on the endogenous levels of Notch activity. Our results indicate that Numb is not a strong inhibitor of Notch activity in the inner ear, and that it has no direct influence upon whether cells become hair cells or supporting cells. However, sustained Numb overexpression seems to impair hair cell differentiation, possibly by interfering with endocytosis or some other process important for hair cell maturation or survival.

## Materials and methods

### Plasmids

We used RT-PCR to isolate from E6 chicken inner ear total RNA the full-length coding sequence of chicken Numb. In mammals, four Numb isoforms can be generated by alternative splicing (Dho et al., 1999; Verdi et al., 1999). From eight individual cloned cDNAs that we isolated by RT-PCR in the chick, one encoded an isoform with a 26 amino acid insert (LPSVIALDLSPLFLQERKFFKGFFGK) in the PTB domain, and was analogous to the human Numb isoform 2 (Verdi et al., 1999), although the chicken insert we isolated is 15 amino-acids longer than the human one and possibly represents a rare splicing variant. The remaining clones encoded an isoform that had no inserts in either the PTB or the C-terminal proline-rich region and resembled both the human Numb isoform 4 and the previously isolated chicken Numb (Wakamatsu et al., 1999). This chicken Numb isoform was almost perfectly identical to the peptide sequence ENSGALP00000015120, the product of the predicted Ensembl chicken *Numb* genomic sequence ENSGALG00000009300. The coding sequence of these two Numb isoforms (cNumb2 and cNumb4) have been deposited in Genbank (KP756695 and KP756696).

The full-length chicken Numb coding sequences were subcloned into RCAS(B) constructs (Morgan and Fekete, 1996), into the bicistronic pIRES2-EGFP expression vector (Clontech), or into Tol2 vectors enabling Tet-ON regulated gene expression (Freeman and Daudet, 2012; Freeman et al., 2012). Plasmid DNA solutions were prepared using a plasmid purification kit (Qiagen, United Kingdom) and diluted for electroporation to 0.8–1 μg/μl in water tinted with Fast Green for visualization.

### Electroporation and retroviral infection of embryonic chick inner ear

Fertile white Leghorn eggs were incubated at 38°C and embryos were staged according to Hamburger–Hamilton (HH) tables. All procedures carried out on chicken embryos were approved by University College London and the UK Home Office. Micro-electroporation of the otic cup with plasmid DNA was performed at HH stages 13–15 (2–2.5 days of incubation) as described in Freeman et al. (2012). After electroporation, eggs were sealed with tape and returned to incubation at 38°C. Some embryos were further treated with Doxycyclin for *in ovo* induction of gene expression at E6 or E7. The numbers of positive embryos analyzed for each type of expression construct were as follows: RCAS-Numb: 35; RCAS-GFP: 12; Numb-IRES2-EGFP: 26; pTRE-Numb-FP635: 20; pTRE-FP635: 8.

### Immunocytochemistry

Embryos were decapitated and their heads immersed in 4% paraformaldehyde in PBS at 4°C for 2–12 h. For whole-mount immunostaining, the membranous part of the inner ear was dissected out from the surrounding cartilage and incubated for 1 h in PBS containing 0.3% Triton X100 and 10% goat serum. All subsequent incubations and rinses were performed in PBS with 0.1% Triton X100 (PBT). Incubations with primary and secondary antibodies were carried out in PBT for 2 h at room temperature or overnight at 4°C. Antibodies and reagents used were: rabbit serum anti-Numb (Wakamatsu et al., 1999, 1/500), rabbit serum anti-GFP (Molecular Probes, 1/2000), mouse monoclonal IgG1 anti-Hair Cell Antigen (HCA, Bartolami et al., 1991, 1/100), mouse monoclonal IgG2a anti-HCS-1/Otoferlin (Gale et al., 2000, 1/100), mouse monoclonal IgG2b anti-TuJ1 (Covance, UK, 1/1000), Alexa 488-, 594-, and 633-conjugated goat IgG secondary antibodies (Molecular Probes, The Netherlands; 1/500 dilution), Alexa 633-conjugated phalloidin (Molecular Probes, 1/100). Nuclei were stained with either DAPI or Syto16. Specimens were mounted in Slowfade (Molecular Probes) and observed under a Zeiss LSM510 confocal microscope. For cryosectioning, embryo heads were fixed as described above, then immersed in a graded series of sucrose-PBS (5-10-20%), embedded in 1.7% agar with 5% sucrose, frozen at −20°C, and cryosectioned at 15 μm thickness.

### Whole-mount *in situ* hybridization

DIG-labeled RNA antisense probes were prepared from a plasmid encoding chick *Hes5.1* (a kind gift of Dr Domingos Henrique). Whole-mount in situ hybridization was performed as in Ariza-Mcnaughton and Krumlauf (2002) with minor modifications. DIG-labeled RNA probes were detected with an anti-DIG peroxidase-tagged antibody (diluted 1:100) and either the TSA-FITC or the TSA-Cy3 amplification system (Perkin Elmer). Following *in situ* hybridization, specimens were processed for Numb or TuJ1 immunostaining as described above.

### Quantification of fluorescence and statistical analysis

Samples co-transfected with the pT2-Hes5::nd2EGFP and either the pTRE-Numb-FP635 or the pTRE-FP635 (control) Tol2 plasmids were fixed for 2 h at room temperature and processed for immunostaining for MyoVIIa/HCA using Alexa 647-conjugated secondary antibody as described above. Confocal stacks (12 bits) were acquired within transfected sensory regions and the mean values of fluorescence intensity for the red (Fluo _Red_) and green (Fluo _Green_) channels within the nucleus of individual cells were collected using ImageJ and the Time Series Analyzer plugin (Schneider et al., 2012). For each confocal stack, mean values and standard deviation (S.D.) for the background levels of red fluorescence (Fluo _Bgd_) were also collected from at least five untransfected cells. Cells with Fluo _Red_ > (Fluo _Bdg_ + 2 × S.D Fluo _Bgd_) were classified as induced cells. In order to compare data across experiments, Z-scoring values for Fluo _Red_ and Fluo _Green_ were computed for all the cells analyzed in each confocal stack. Statistical analysis of the data and graphs were made with the OriginPro 9.1 software (OriginLab Corporation).

## Results

### Numb is expressed in dividing progenitor cells in the chick inner ear

**Figure 1:**
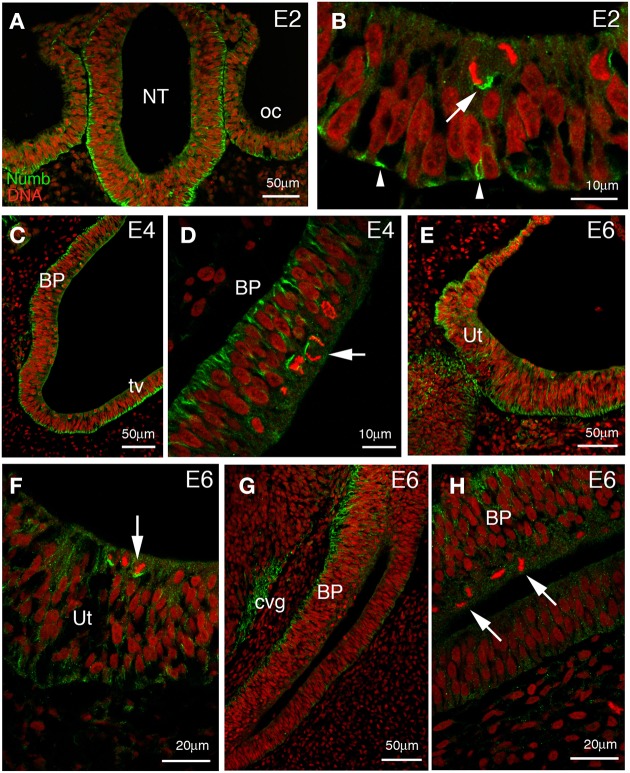
**Numb expression at early stages of chick inner ear development**. Transverse sections through the hindbrain and parallel to the future longitudinal axis of the cochlea. **(A)** Low power picture at 2 days of incubation (E2) showing the hindbrain and adjacent otic vesicles. Numb is expressed throughout the otic epithelium and neuroepithelium. **(B)** Higher power views of the otic epithelium. The staining is predominantly basal (arrowheads) and remains on the basal side of cells rounded up for mitosis. Note the asymmetric division (arrow) where Numb is preferentially segregated into one of the two daughter cells. **(C)** At E4, Numb is expressed in all of the cells of the future basilar papilla (BP), and also in the non-sensory region (presumptive tegmentum vasculosum, tv). Basal Numb crescents can be seen in mitotic cells at the apical surface. **(D)** Detail of the same future basilar papilla, showing basal Numb crescents in mitotic cells (arrow). **(E)** Developing utricle (Ut) at E6. Numb is still expressed throughout the epithelium and is basal. **(F)** Detail of **(E)**; in the majority of utricular mitotic cells, basal Numb crescents can still be seen (arrow). **(G)** Developing basilar papilla (BP) at E6. Numb is basally localized in the epithelium, and is also seen in the cochleovestibular ganglion (cvg). **(H)** Detail of **(G)**; no Numb crescents are detected in the mitotic cells here (arrows).

The inner ear develops from the otic placode, a thickening of the head ectoderm visible from stage 10 (36 h of incubation) in the chick embryo. The placode invaginates to first form a cup, then a hollow epithelial sphere named the otic vesicle. At these early stages, Numb protein is detected throughout the otic epithelium, in both presumptive sensory and non-sensory regions (Figures 1A,B). As in the neural tube, Numb is expressed in the majority of cells and is strongly localized to the basal surface. At mitosis, when a cell moves its nucleus to the apical surface, Numb forms a characteristic crescent on the more basal side of the cell. This persistent basal localization of Numb is independent of the orientation of the cleavage plane. We found that cleavage planes were quite variable: some were perpendicular to the plane of the epithelium (“vertical”), producing two cells that lay side by side in the epithelium; others were tilted (though rarely horizontal) such that a division produced one cell more apically and one cell more basally (Figure 1B). Because Numb is always basal, a vertical (symmetrical) cleavage will presumably entail that both daughters inherit Numb, while in a tilted (asymmetrical) cleavage only the basal daughter will do so.

As the otic vesicle grows, parts of the epithelium become specialized to form several sensory patches: three cristae and three maculae (the utricle, saccule, and the macula neglecta) in the vestibule, for balance; the basilar papilla, in the cochlea, for hearing; and the lagenar macula, at the tip of the cochlea, for balance. Within each sensory patch, progenitor cells divide repeatedly and give rise to hair cells and supporting cells. Both these differentiated cell types can arise from the same type of progenitor cell (Fekete et al., 1998; Stone and Rubel, 1999; Lang and Fekete, 2001), and the choice of cell fate depends on lateral inhibition mediated by Notch signaling, as discussed earlier. Thus, if asymmetric distribution of Numb influences the choice of cell fate, one might expect that Numb should be present in dividing progenitors at the time of the terminal mitoses that give rise to hair cells. In the vestibular patches, where hair cells begin to be born at E4, Numb crescents were indeed visible at this time (not shown), but they had become less prominent by E6 (3/9 mitotic cells from three separate embryos; Figures 1E,F) although hair cell production is then still in progress. In the basilar papilla, where hair cells are born at E5–E8 (Katayama and Corwin, 1989; Bartolami et al., 1991), Numb crescents were present at E4 (Figures 1C,D) but could no longer be detected at all in mitotic cells at E6 (12 mitotic cells from three separate embryos; Figures 1G,H).

**Figure 2:**
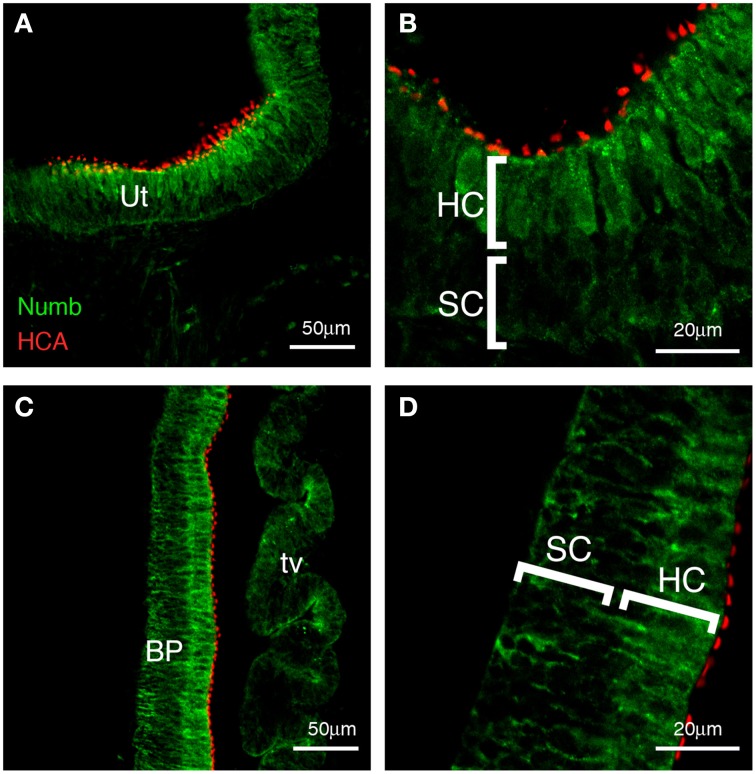
**Numb expression at late stages of chick inner ear development**. Transverse sections (E10), showing expression of Numb (green) and HCA, marking the stereociliary bundles of hair cells (red). **(A,B)** Vestibular patches and **(C,D)** basilar papilla. Strong Numb expression is found only in the mature hair cells, and within these cells it is no longer localized but is diffuse throughout the cytoplasm. In the supporting cells (SC), Numb is absent or greatly reduced. Any Numb in these cells is basally located. Ut, utricle; BP, basilar papilla; HC, hair cells; SC, supporting cells; tv, tegmentum vasculosum.

By E7 many hair cells have differentiated in the vestibular patches, and a few hair cells can be detected in the basilar papilla. At this time, Numb expression is concentrated within the hair cells and it no longer has a basal location. Instead, it is expressed diffusely throughout the cytoplasm. This intracellular localization is consistent with previous reports indicating that Numb interacts with components of the endocytotic machinery (Offenhauser et al., 2000; Santolini et al., 2000; Berdnik et al., 2002; Nishimura et al., 2003). Within the supporting cells, there is no strong Numb expression, although some weak staining is apparent at the basal edge of the epithelium (not shown). At E10, this expression pattern is maintained in vestibular patches (Figures 2A,B), and it becomes more apparent in the basilar papilla (Figures 2C,D), which now has its full complement of hair cells (Katayama and Corwin, 1989). These results suggest that the hair cells and not the supporting cells either inherit Numb or switch on its expression at an increased level. The diffuse distribution of Numb in the hair cells persists in both vestibular and auditory patches until at least E12.

These observations leave open the possibility that high levels of Numb drive cells toward a hair-cell fate, possibly by an effect on Notch signaling.

### Sustained Numb overexpression does not promote commitment to a hair-cell fate

Several Numb isoforms have been reported in vertebrates (Dho et al., 1999; Verdi et al., 1999; Karaczyn et al., 2010), and we identified by RT-PCR two splice variants of the Numb gene in the E6 chicken inner ear, differing by the presence of a 26 amino-acid insert within the PTB domain (see Materials and Methods). These two isoforms exhibited the same membranous and vesicular subcellular localization in transfected chicken embryonic fibroblasts (data not shown) and produced an identical phenotype in our gain-of-function experiments, hence for clarity we will hereafter refer to either of these isoforms as Numb.

To test whether Numb indeed regulates commitment to a hair-cell fate, we investigated the consequences of overexpressing Numb in the developing chick inner ear. To overexpress Numb, we first used an RCAS-Numb proviral DNA that was transfected in the otic cup at stage HH 13–14 (E2). As a control, some specimens were electroporated with RCAS-GFP plasmid DNA.

Following electroporation, the inner ear epithelium showed persistent long-term expression of the transgene carried by the plasmid—GFP or exogenous Numb as the case might be—as judged by anti-GFP or anti-Numb immunostaining. This was visible from 24 h until at least 10 days after electroporation (data not shown). The duration and extent of overexpression suggest that viral DNA had become integrated, giving rise to a spreading infection. Thus, we refer below to “infected” specimens even though RCAS plasmid electroporation, rather than RCAS viral particles, was used to initiate the process.

**Figure 3:**
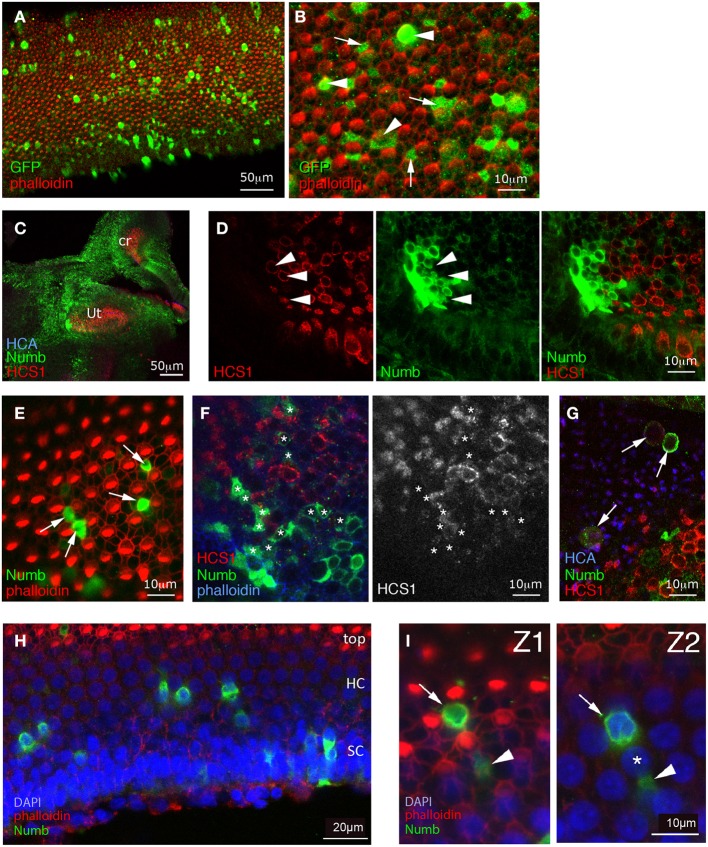
**Effects of sustained Numb overexpression using retroviral vectors**. Specimens infected at E2 with RCAS-GFP **(A,B)** or with RCAS-Numb **(C–I)**, and fixed at 8 days post-electroporation (E10). **(A,B)** RCAS-GFP infected samples. Surface views of the basilar papilla show GFP expression in scattered cells. At high magnification, many of these GFP positive cells are seen to be hair cells (arrowheads), while others are supporting cells (arrows). **(C)** Low magnification view showing strong Numb overexpression (green) throughout the inner ear sensory and non-sensory regions (Ut, utricle; cr, crista). **(D)** Detail of the utricle shown in **(C)**, with a patch of infected cells overlapping the sensory patch. Within the infected patch, there are some HCS1-positive hair cells (arrowheads) but none of these are overexpressing Numb. **(E)** In the basilar papilla, the cells overexpressing Numb (arrows) lack hair bundles and occupy positions characteristic of supporting cells, intercalated between hair cells. **(F)** Infected sensory crista, seen in a confocal optical section passing obliquely through the hair cell layer. As in other sensory patches, the Numb-infected cells (asterisks) do not express hair-cell markers such as HCS1. **(G)** An infected utricle. Some of the Numb-infected cells lie above the surface of the epithelium (identified by the HCA staining) and appear to have been extruded from it. These cells have rounded cell bodies, and they display a faint HCS1 immunoreactivity (arrows). **(H)** A basilar papilla with Numb infected cells (green), seen in an oblique optical section. The apical most region of the epithelium is at the top of the micrograph (with the hair cell stereocilia visible, in red), and the basal region is at the bottom, and contains the supporting cell nuclei. Note that the apical, hair cell nuclei are regularly interspaced, and display less DAPI staining than the basal, supporting cell nuclei. **(I)** High magnification view of the specimen shown in **(H)**; Z1 corresponds to a more apical optical section than Z2. Some Numb-overexpressing cells have typical supporting cells morphologies (arrowheads) and extend their thin cellular processes in between the hair cells nuclei (asterisks), while some other (arrow) have a rounded appearance and a nucleus located in the hair-cell nuclei layer. The latter may correspond to hair cells that have failed to differentiate correctly. In the basilar papilla, they represent about 15% of the Numb-infected cells.

We took specimens at E10, 8 days after electroporation, and co-immunostained them for GFP and hair cell markers—either Hair-Cell Antigen (HCA, a protein tyrosine phosphatase receptor Q) (Bartolami et al., 1991; Goodyear et al., 2003) detected in the stereociliary bundle, or HCS1/otoferlin (Goodyear et al., 2010) expressed in the hair-cell cytoplasm. In the control specimens infected with RCAS-GFP, we saw GFP in both hair cells (approximately 25% of infected cells in the basilar papilla, *n* = 4 specimens analyzed) and supporting cells (Figures 3A,B). The levels of GFP immunostaining were quite variable from one cell to the other, but the differentiation of both hair cells and supporting cells appeared unaffected by the infection.

In RCAS-Numb infected specimens, the overall morphology of the inner ear was normal. Sixteen such specimens were examined at 8 days after electroporation, and 10 of these contained groups of cells or scattered cells overexpressing Numb protein within the sensory patches; large patches of infection were also frequently found outside the sensory patches (Figure 3C). The levels of expression of exogenous Numb protein were much higher than the levels of endogenous Numb in non-infected cells, and infected cells could be identified readily. The exogenous Numb was present at the cell membrane and in the cytoplasm, but not asymmetrically localized or concentrated at the basal ends of the infected cells. We looked closely at the cells overexpressing Numb within sensory patches, and we found, to our surprise, that none of them expressed the HCA or HCS1 hair-cell marker (Figures 3D–F). In the utricle of one specimen, we could find RCAS-Numb infected cells expressing HCS1 and HCA at a very low level, but with their cell bodies lying at the surface of the epithelium as if they had been expelled from it (Figure 3G). The majority of Numb-positive cells (88% out of over 140 infected cells counted in four different specimens) looked like typical supporting cells, intercalated between hair cells. A minority (12%) of infected cells, however, in both auditory and vestibular sensory patches, had a more cylindrical shape than typical supporting cells and had nuclei located at a more apical level than normal for supporting cells (Figures 3H,I).

From this we conclude that cells overexpressing Numb, far from becoming hair cells as predicted by the original hypothesis, predominantly become supporting cells: this is the most frequent fate, at least for cells that do not die and disappear from the epithelium. The minority that do not resemble normal supporting cells do not become normal hair cells either: they either fail to display normal hair-cell molecular markers, or express those markers weakly or seem to be extruded from the epithelium.

### Early vs. late overexpression of Numb differentially affect hair cell differentiation

The absence of hair cells overexpressing Numb could have various causes: Numb could, contrary to the initial hypothesis, block commitment to a hair-cell fate; hair cells might switch off expression of virus-encoded Numb so much more efficiently than supporting cells that they appear uninfected; the exogenous Numb might cause hair cells to die; or Numb overexpression might leave commitment unaffected, but block the subsequent process of differentiation. To distinguish among these possibilities, and to test for the effects of Numb at distinct stages of inner ear development, we used different vectors enabling either an early or a late pulse of Numb expression.

**Figure 4:**
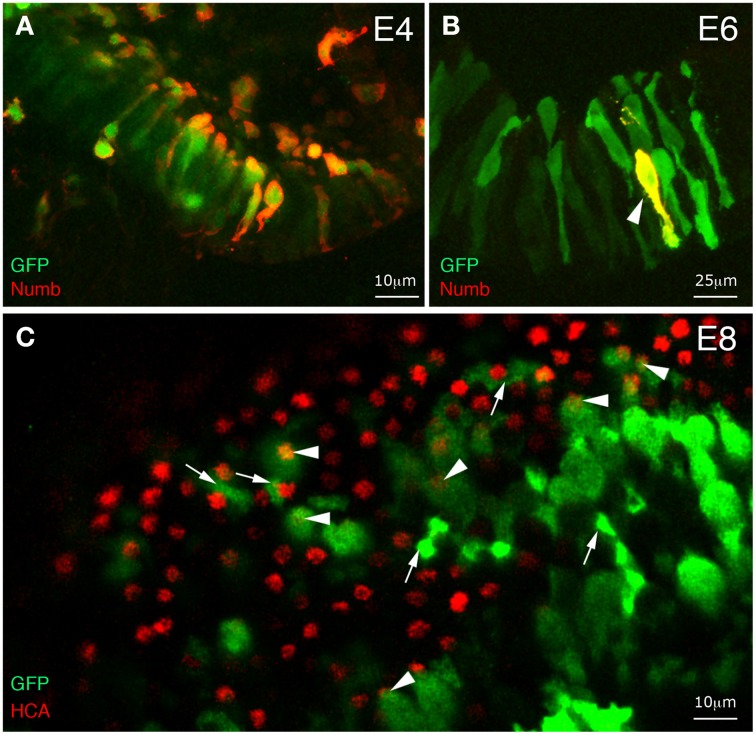
**Effects of early and transient Numb protein expression on hair cell differentiation**. Whole-mount immunostaining of inner ear tissue from chick embryos electroporated at E2 with Numb-IRES2-EGFP. **(A)** At 2 days post-electroporation, most GFP-positive cells coexpress Numb. **(B)** At 4 days post-electroporation, only a few transfected cells maintain a strong expression of Numb (arrowhead). **(C)** Surface view of an utricular macula at 6 days post-electroporation. Some transfected cells differentiate into hair cells (arrowheads) and others into supporting cells (arrows).

To test the effect of overexpressing Numb transiently and at a time when the first hair cell fate decisions occur, we electroporated the otic epithelium at E2 with a bi-cistronic Numb-IRES2-EGFP expression plasmid, driving conjoint expression of Numb and EGFP. Although the plasmid functions only transiently in the transfected cells, the GFP protein is very stable, thus allowing us to identify the transfected cells up to a week later using GFP immunostaining. We followed the time-course of Numb expression in these cells by immunostaining for Numb. At 2 days post-electroporation (E4), when cells in the vestibular patches are just beginning to undergo terminal mitosis and to become committed to a hair-cell fate, the majority of GFP-positive cells showed strong Numb expression (Figure 4A). However, at E6, almost all of them had ceased to overexpress Numb (Figure 4B), suggesting that production of Numb protein from the plasmid had ceased sometime between 2 and 4 days post-electroporation. In spite of the overexpression of Numb during the critical period for cell fate decisions, we saw no tendency of Numb-IRES2-EGFP transfected cells in the vestibular patches to differentiate into hair cells rather than supporting cells: at E8, approximately 22% of the GFP-positive cells (out of *n* = 290 cells analyzed in seven different specimens) had differentiated into hair cells and 78% into supporting cells (Figure 4C). Thus, the two cell fate choices seemed to have been made with the normal relative probabilities, and to have been followed by normal differentiation of both cell types.

**Figure 5:**
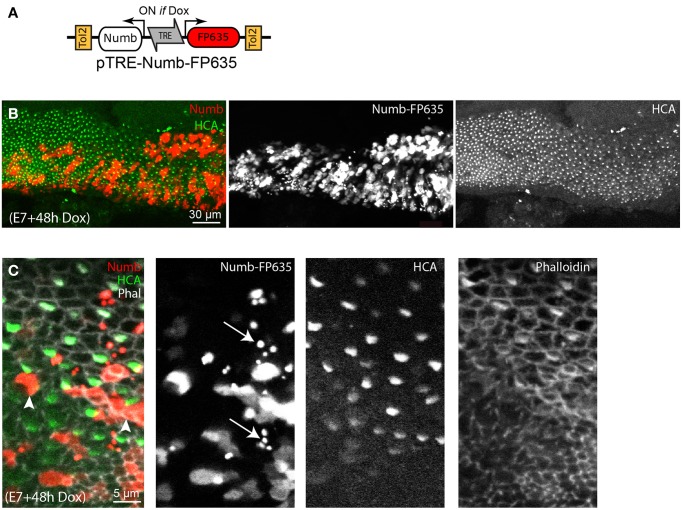
**Effects of conditional Numb overexpression at stages of hair cell differentiation. (A)** Schematic representation of the pTRE-Numb-FP635 Tol2 Tet-on construct driving expression of Numb and the red fluorescent protein FP635 following Dox treatment *in ovo*. **(B)** Basilar papilla analyzed 48 h after Dox treatment at E6. A large number of Numb-induced cells, identified by FP635 expression, are present within the sensory epithelia. **(C)** High magnification view of the sample shown in **(B)**. None of the Numb-FP635 induced cells express HCA (arrowheads). Note also the presence of small vesicle-like structures with strong FP635 fluorescence (arrows) within transfected regions.

In order to test further the effects of Numb on late hair cell differentiation, we next used a Tol2 transposon vector enabling conditional induction of gene expression upon doxycycline (Dox) treatment (Figure 5). This construct, pTRE-Numb-FP635, contains a bi-directional promoter enabling co-expression of Numb and the red fluorescent protein Turbo-FP635 (FP635 thereafter) in order to identify induced cells (Figure 5A). Embryos transfected at E2 with the Tol2 transposase, a Tol2 plasmid driving expression of the rtTA “Tet-ON” transactivator, and pTRE-Numb-FP635 plasmids were left to develop until E7, at which point they were treated *in ovo* with 10 μg of Dox. The embryos were left to develop a further 48 h before their inner ear tissue was processed for immunostaining with Myo7A (expressed in hair cell cytoplasm) and HCA antibodies. The Numb-induced samples exhibited a phenotype very similar to that resulting from RCAS-Numb electroporation: none of the Numb-induced cells (identified by FP635 expression) expressed hair cell markers (Figures 5B,C). Furthermore, we noticed the presence of numerous cellular debris with strong FP635 fluorescence within transfected regions (Figure 5C), an observation further reinforcing the hypothesis that Numb overexpression causes cell death. From these data, we conclude that a transient and early pulse of Numb expression has no impact on hair cell vs. supporting cell fate decisions, but elevated levels of Numb expression could have a detrimental effect on hair cell survival.

### Numb overexpression does not strongly inhibit Notch activity in the ear

**Figure 6:**
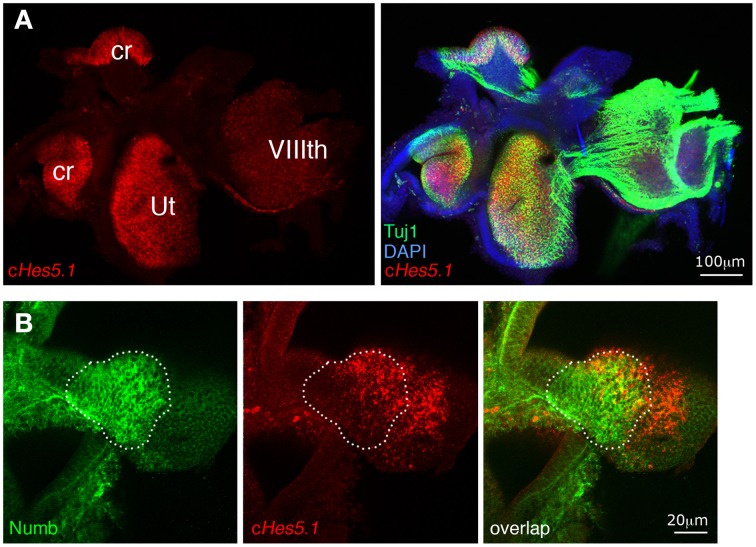
**Sustained Numb overexpression does not affect *Hes5.1* expression. (A)** The Notch target gene *Hes5.1* (in red) is strongly expressed within inner ear sensory patches (ut, utricle; cr, crista). Beta-III tubulin (TuJ1) immunoreactivity (green) is intense in nerve fibers originating from the VIIIth ganglion and within sensory patches, coincident with the *Hes5.1* signal. **(B)** An RCAS-Numb infected sensory crista. Note that the levels of *Hes5.1* expression are unchanged in the infected region (dotted line) relative to the uninfected region, and that overexpression of Numb does not induce expression of *Hes5.1* outside of the sensory patch epithelium.

The preceding results imply that Numb does not control cell fate choices in the way that our original hypothesis would predict, but it remains possible that Numb regulates the activation of the Notch pathway. To find out, we first investigated the effects of exogenous Numb on expression of *Hes5.1*, a member of the *Hairy and Enhancer of Split* (*Hes*) family of genes, whose transcription is regulated by Notch activity and which thus serve as reporters of Notch activation in a number of tissues (Kageyama and Ohtsuka, 1999). In the mammalian cochlea and vestibule, *Hes1* and *Hes5* are expressed by supporting cells and their inactivation in knock-out mice results in overproduction of hair cells (Shailam et al., 1999; Zine et al., 2001; Tateya et al., 2011). The chicken *Hes5.1* gene is an ortholog of the mammalian *Hes5* gene, and appears to be functionally equivalent (Fior and Henrique, 2005; Abello et al., 2007). In the developing inner ear, it is first expressed within the anterior region of the otic cup where neuroblasts delaminate, then within all inner ear sensory patches at the time of hair cell production (Figure 6A and Daudet et al., 2007).

If Numb blocks Notch activity as hypothesized, its overexpression should cause loss of *Hes5.1* expression in the infected cells. To test this, we analyzed by fluorescent *in situ* hybridization *Hes5.1* expression in inner ear sensory patches infected with RCAS-Numb and fixed at E6 and E8. At both stages, we found patches of infected cells (*n* = 8 infected patches in five embryos) located within (*n* = 5) or abutting (*n* = 3) sensory patches. As in the adjacent uninfected regions, some of these cells expressed *Hes5.1* and others did not; there was no obvious difference between the infected and uninfected regions, neither in the proportion of cells expressing *Hes5.1* nor in their levels of expression of *Hes5.1* (Figure 6B). This result suggests that Numb is not a negative regulator of Notch activity in the inner ear, but it remained difficult to precisely quantify at the cellular level the potential effects of Numb on Notch activity using *in situ* hybridization.

**Figure 7:**
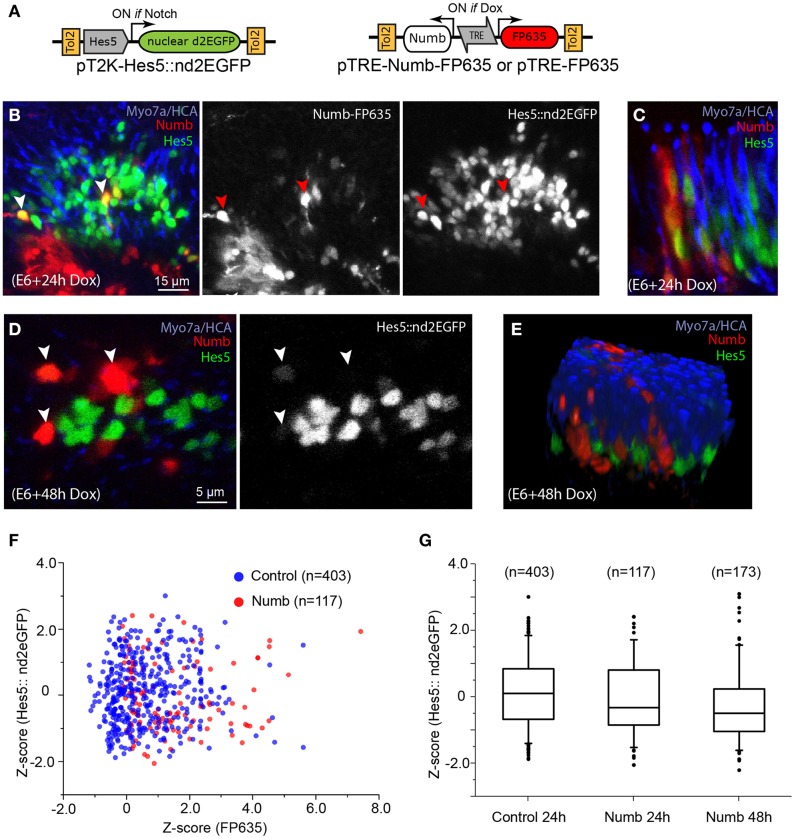
**Numb overexpression has a mild inhibitory effect on Notch activity. (A)** Schematic representation of the Tol2 Numb-inducible construct and the Hes5 reporter used for the experiments. **(B)** Whole-mount preparation of a sensory crista with Numb-induced cells 24 h after Dox treatment. The sensory epithelium contains a large number of cells with variable levels of Hes5::nd2EGFP fluorescence in their cytoplasm. Note that some of the Numb-induced cells have high levels of Hes5 reporter fluorescence (red arrowheads), comparable to those of their neighboring, uninduced cells. **(C)** Another example of Numb-induced cells with Hes5 reporter fluorescence in their nuclei, seen from a transverse aspect. **(D)** At 48 h after Dox treatment, the Numb-induced cells appeared to have less Hes5 reporter fluorescence in their nuclei compared to non-induced cells but do not express the hair cell marker Myo7A. **(E)** A 3D rendering of the confocal stack from which the optical slice shown in **(D)** was selected, demonstrating that none of the Numb-induced cells expressed hair cell markers such as HCA or Myo7A (see also Movie S1). **(F)** Scatter plot of the Z-score for FP635 and Hes5::nd2EGFP fluorescence levels for individual cells positive for FP635 expression (see Methods) 24 h after Dox treatment, for control (FP635 alone) or Numb-FP635 induced cells. Note the absence of correlation between both parameters. **(G)** Box plot (box: 25–75% of cells, whiskers: 5–95% of cells, and dots: outliers) of the FP635-positive cells positive for control (24 h post-Dox), and Numb-induced conditions at 24 and 48 h after Dox treatment.

To test further the cell-autonomous effects of Numb on Notch signaling, we next used a Tol2 vector encoding a reporter of Notch activity, pT2-Hes5::nd2EGFP (Chrysostomou et al., 2012). In cells transfected with this construct, expression of a nuclear-localized and destabilized green fluorescent protein is regulated by the mouse Hes5 promoter (Takebayashi et al., 1995), providing a direct readout of the endogenous levels of Notch activity. In a previous study, we showed that this reporter is active within sensory progenitors and supporting cells of the chicken inner ear, and that it exhibits a rapid reduction in fluorescence levels following pharmacological blockade of Notch activity by the gamma-secretase inhibitor DAPT (Chrysostomou et al., 2012) or following induction of Sox21 expression (Freeman and Daudet, 2012). We co-transfected the chicken inner ear *in ovo* with the Hes5::nd2eGFP reporter and Dox-inducible Tol2 vectors driving expression of Numb-FP635, or FP635 alone as a control (Figure 7A and Figure S1). The embryos received Dox at E6, and their inner ear were fixed 24 or 48 h later then immunostained for HCA and Myo7A. The Hes5 reporter was active in cells with typical supporting cell and progenitor cell morphology, but not in differentiated hair cells (Figure 7B and data not shown). Probably owing to the mosaicism of transfection, only a subset of Hes5-positive cells were also FP635-positive in both control and Numb-induced cells. At 24 h post-induction, there was an important variability in the levels of Hes5::nd2EGFP fluorescence in the nucleus of both control (Figure S1) and Numb-positive cells, some of which clearly had high levels of Hes5 reporter fluorescence (Figures 7B,C,F). In contrast, at 48 h the levels of Hes5::nd2EGFP fluorescence appeared reduced in the majority of Numb-induced cells compared to non-induced cells (Figures 7D,E; Movie S1). A quantitative analysis of Hes5 reporter fluorescence in single (induced) cells (see Materials and Methods) showed that at both 24 and 48 h post-induction, there was a small reduction in the levels of Hes5::nd2EGFP fluorescence in Numb-induced cells compared to control, FP635-induced cells (Figure 7G). This reduction was not significant at 24 h (Mann–Whitney *U* = 25981; *p* = 0.09278) but significant at 48 h (Mann–Whitney *U* = 43494; *p* = 2.07E-6). We conclude from these results that Numb overexpression does induce a reduction in the intrinsic levels of Notch activity in the inner ear, but this effect is relatively mild and slow to develop, and Numb is therefore unlikely to significantly contribute to the regulation of hair cell fate decisions.

## Discussion

Numb was identified in Drosophila as a critical regulator of Notch-dependent cell fate decisions during neurogenesis, acting as cell-intrinsic inhibitor of Notch activity during lateral inhibition. Although the vertebrate homologs of Numb were later proposed to have a similar function in vertebrates, some data suggested that they could also affect additional cellular processes. In this study, we set out to investigate Numb function in the embryonic chick inner ear using gain-of-function approaches. Our findings suggest that Numb is not a critical determinant of hair cell fate decisions and does not prevent the reception of lateral inhibition. Furthermore, high levels of Numb expression interfere with normal hair cell differentiation, an observation that hints at non-Notch dependent functions for Numb in the ear.

### Numb does not bias hair cell fate decisions in the inner ear

The molecular mechanisms regulating mechanosensory cell formation are evolutionary conserved. The vertebrate homolog of Atonal (Atoh1), a proneural bHLH transcription factor essential for the differentiation of the mechanosensory cells in flies, is required for hair cell differentiation in the inner ear (reviewed in Jarman and Groves, 2013). Furthermore, lateral inhibition mediated by the Notch pathway regulates cell fate diversification within the sensory organ precursor (SOP) lineage of *Drosophila* and in the inner ear (Eddison et al., 2000). Asymmetric cell division (reviewed in Zhong and Chia, 2008), which generates two daughter cells with unequal developmental potential at birth, is also an important contributor to cell diversification in the SOP lineage. Numb is one of the intrinsic factors asymmetrically inherited by one of the daughter cells, in which it inhibits Notch activity. As a consequence, the Numb-expressing cell gains a competitive advantage and is more able to deliver lateral inhibition to its sibling. In vertebrates, Numb has been proposed to have a similar function during neurogenesis (Wakamatsu et al., 1999; Shen et al., 2002; McGill and McGlade, 2003; Matsuda et al., 2005), where asymmetric cell divisions are thought to contribute at least partly to the control of neural differentiation.

In the embryonic chick inner ear, we found that Numb is initially expressed by all epithelial cells, including sensory progenitors, and analysis of fixed tissue suggest that Numb can be distributed asymmetrically in some dividing cells, as previously reported for neural progenitor cells. Numb then becomes enriched in differentiated hair cells, which have escaped Notch activation in order to commit to a hair cell fate. Our results, however, argue against the idea that Numb acts as a determinant of hair-cell fate decisions, either by blocking activation of Notch, or indeed by any other mechanism. Cells overexpressing Numb at either early or late stages of development are not biased toward a hair-cell fate, and they do not show altered expression of the Notch effector *Hes5*.1.

Using a genetically encoded fluorescent Hes5 reporter to quantify more precisely the effects of Numb on Notch activity, we found that Numb-induced cells do exhibit a significant reduction in the Hes5 reporter fluorescence levels 48 h after Dox treatment. Although this effect might suggest an interference with the reception of Notch signaling, several observations conflict with that interpretation. Firstly, the reduction in Hes5 reporter fluorescence in Numb-induced cells was very variable from cell to cell and did not appear to be correlated to the levels of expression of the Numb transgene in individual cells. Secondly, there was no significant reduction in the levels of Hes5 reporter fluorescence 24 h after Numb induction. In our previous work, we found that inhibition of Notch activity with DAPT led to an almost complete extinction of the same Hes5 reporter in less than 12 h (Chrysostomou et al., 2012), and Sox21 induction *in ovo* can result in a much stronger inhibition of this Hes5 reporter (Freeman and Daudet, 2012). By comparison, the reduction of Hes5 reporter fluorescence levels in Numb-induced cells is relatively modest and slow to develop, suggesting that Numb is neither directly nor strongly inhibiting Notch activity. Finally, the majority of cells transfected with Numb differentiated into supporting cells, and not hair cells as would be expected from a strong inhibition of Notch signaling. Altogether, these data suggest that Numb has little impact on intrinsic levels of Notch activity and is unlikely to act as a critical regulator of hair cell fate decisions. The reduction in Hes5 reporter fluorescence observed 48 h after Numb induction may reflect a weak and an indirect effect of Numb on Notch activity, but it may also be consecutive to some unspecific effect on the processing of the fluorescent reporter itself or some other aspect of cell metabolism that remains to be determined.

One surprising observation is that none of the Numb-overexpressing cells expressed hair cell markers, suggesting that Numb misexpression could interfere with normal hair cell differentiation and survival. A small fraction of the Numb-overexpressing cells also exhibited a mis-positioned nucleus in between the hair cell and supporting cell layers, suggesting a potential defect in their apico-basal polarity. It is possible that these merely reflect a non-specific toxic effect of the overexpressed protein, but Numb certainly has documented functions in other systems that might account for these abnormalities. In particular, Numb has been implicated in endocytosis (Santolini et al., 2000; Berdnik et al., 2002; Nishimura et al., 2003) as well as the maintenance of cell adhesion and apico-basal polarity (Rasin et al., 2007; Sato et al., 2011), and disturbance of both processes could interfere with hair-cell maturation. Numb is also expressed in terminally differentiated neurons (Zhong et al., 1997; Dooley et al., 2003), and it seems to be involved in neuronal maturation (Klein et al., 2004) and axonal growth (Nishimura et al., 2003; Huang et al., 2005). As it has been suggested in the context of neurogenesis, the changing localization of Numb in the developing ear could indicate that it may have different functions at different stages. Although we have not directly investigated these possibilities, Numb could have a role in the regulation of otic neurogenesis or cell proliferation. Numb functions, however, do not appear to include regulation of Notch activity in the context of hair cell fate decisions. One important caveat should be mentioned, however: we have performed overexpression experiments only, and have not been able to test the effects of eliminating Numb function in the chick inner ear. It is conceivable that a complete absence of Numb might alter Notch-regulated cell fate decisions, even though Numb overexpression does not do so.

### The functions of Numb in the vertebrate nervous system cannot be explained simply in terms of Notch regulation

Our findings add to the questions surrounding the proposed role of Numb as an asymmetrically inherited regulator of Notch activity in other vertebrate tissues, especially in the nervous system. As in *Drosophila*, neuroepithelial progenitor cells of the central nervous system of vertebrates can divide along different cleavage planes, and some studies suggest that the cleavage plane can be linked to the assignment of asymmetric daughter cell fates (Chenn and McConnell, 1995; Cayouette and Raff, 2003; Das et al., 2003; Das and Storey, 2012), although there is still some disagreement about this (Konno et al., 2008; reviewed in Zhong and Chia, 2008). There is, on the other hand, very strong evidence that a cell with Notch activated remains as a neural progenitor, whereas a cell that escapes Notch activation differentiates as a neuron. It is an obvious suggestion, therefore, that if there is a link between cell fate and the orientation of cell division, it could depend on an asymmetrically distributed factor that is inherited by the prospective neuron and inhibits Notch activity.

This notion was encouraged by the finding that vertebrate homologs of Numb are expressed by neural progenitor cells in chick and mammals (Zhong et al., 1996; Wakamatsu et al., 1999). However, despite a number of studies in both organisms, the role of Numb in vertebrate neurogenesis remains controversial. This is due in part to the fact that the subcellular localization of Numb in dividing neuroepithelial progenitors appeared to vary, depending on the species. In mice, it was initially reported that Numb accumulates at the apical pole of neuroepithelial cells and forms crescent-like structures in mitotic cells, and is therefore likely to be inherited by the cell that remains as a progenitor in the lumenal region of the neural tube after an apico-basal cell division (Zhong et al., 1996, 1997; Petersen et al., 2002, 2004). However a subsequent study reported that at least in the developing cortex, Numb is in fact enriched at the apical end-feet of interphase radial glial cells that are tightly surrounding mitotic cells (Rasin et al., 2007). In the chicken, Numb is clearly localized on the baso-lateral side of neuroepithelial cells in the neural tube, implying that after an apico-basal cell division it would be predominantly inherited by the basally located daughter cell, which is thought to migrate away from the proliferative zone and differentiate into a neuron (Wakamatsu et al., 1999). Thus, it has been proposed that in mice, Numb causes cells that inherit it to remain as progenitors, whereas in chick, it has been suggested that it has an opposite effect, causing them to differentiate as neurons.

Studies on cultured mouse cortical progenitor cells and in the mouse retina have demonstrated an involvement of Numb in asymmetric cell division, but with complex effects, including localization of Numb to the neuronal daughter cells in some circumstances (Cayouette et al., 2001; Shen et al., 2002; Cayouette and Raff, 2003). Mice with knockout mutations of *numb* show abnormalities of cortical and sensory neurogenesis (Zhong et al., 2000) and sensory neuron production (Zilian et al., 2001), but the mutant animals die at E11.5 from defects in neural tube closure. The early lethality can, however, be bypassed by means of conditional knockouts, which have been used to delete simultaneously both *numb* and the closely related *numblike* gene in the embryonic forebrain. Studies using different drivers for this double conditional knockout reported surprisingly different results, with apparently opposite effects on cell fate choices (Petersen et al., 2002, 2004; Li et al., 2003). Both sets of observations share one striking feature, however: the cytoarchitecture of the neural tube is disrupted and the dividing progenitors fail to stay in their proper location, in the ventricular zone. Likewise, deletion of *numb* and *numblike* in the embryonic retina leads to abnormal proliferation and severe defects in epithelial organization (Kechad et al., 2012).

Perhaps, therefore, the fundamental function of Numb in the vertebrate neuroepithelium is not to control cell fate decisions through effects on Notch signaling, but rather to control epithelial architecture through effects on cell polarity, cell adhesion, and the cytoskeleton. Indeed, there is growing evidence that Numb can have other functions apart from influencing cell fate choices: for example, mice with conditional knockout mutations of *numb* and *numblike* in their sensory ganglia have no deficit of neurogenesis, but show defects in axonal arborization (Huang et al., 2005). The different Numb isoforms can bind to a number of proteins of the endocytotic machinery (Krieger et al., 2013), hence the functions of Numb are expected to be highly context-dependent, affecting the intracellular trafficking of Notch receptors but also that of any other receptor or cell surface molecule directly or indirectly binding to Numb. Our findings are consistent with the view that Numb influences primarily cell structure and maturation rather than Notch-dependent cell fate choices in the inner ear. And if commitment to a hair-cell fate is after all regulated by an asymmetrically distributed factor that interferes with Notch signaling, that factor is not likely to be Numb.

### Conflict of interest statement

The authors declare that the research was conducted in the absence of any commercial or financial relationships that could be construed as a potential conflict of interest.
