# Supplementary figures and images for: Numb is not a critical regulator of Notch-mediated cell fate decisions in the developing chick inner ear
Source: Front Cell Neurosci. 2015 Mar 12;9:74. doi: 10.3389/fncel.2015.00074 (PMC4357303; doi:10.3389/fncel.2015.00074)

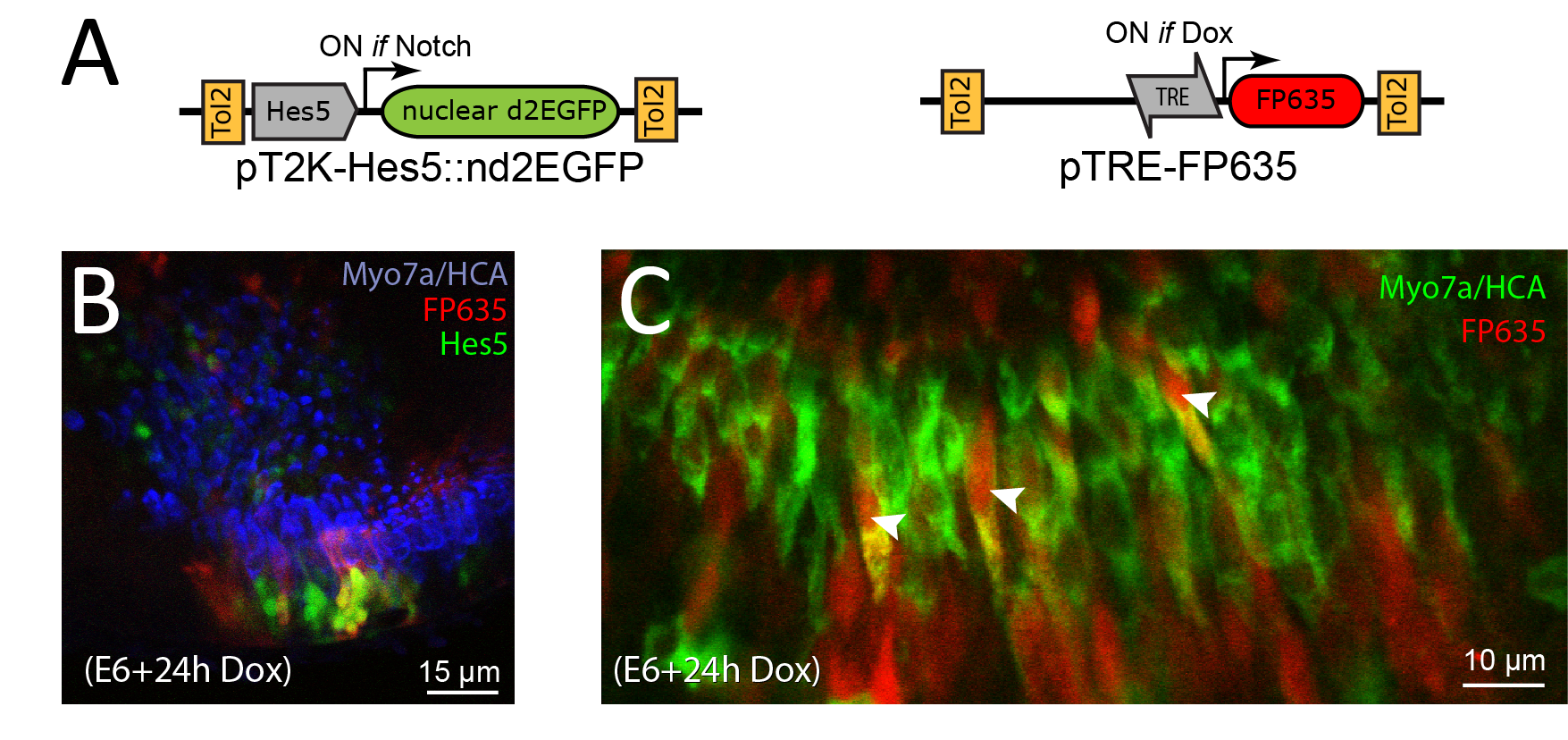

Supplement: Supplementary file 2 [file Image1.TIF]
